# Senescent macrophages alter fibroblast fibrogenesis in response to SARS-CoV-2 infection

**DOI:** 10.1515/nipt-2022-0003

**Published:** 2022-03-25

**Authors:** Brandt Pence, Yufeng Zhang, Ivy Antwi, Theodore James Cory

**Affiliations:** University of Memphis College of Health Sciences, Memphis, TN, USA; Department of Clinical Pharmacy and Translational Science, University of Tennessee Health Science Center College of Pharmacy, Memphis, TN, USA

**Keywords:** fibroblast, fibrogenesis, macrophage, SARS-CoV-2, senescence

## Abstract

SARS-CoV-2 has, since its emergence in 2019, become a global pandemic. Disease outcomes are worsened in older patients who are infected. The causes for this is multifactorial, but one potential cause for this disparity is increased rates of cellular senescence in older individuals, particularly in immune cells. Cellular senescence, the accumulation of factors resulting in cell growth arrest and apoptosis resistance, increases as individuals age. In immune cells, senescence is associated with increased inflammation, and alterations in immune response. We utilized a co-culture system consisting of senescent or non-senescent macrophages directly cultured with fibroblasts, and infected with SARS-CoV-2. We assessed the expression of collagen and fibronectin, important molecules in the extracellular matrix, as well as a number of fibrogenic factors. We observed that infection with SARS-CoV-2 induced collagen production in co-cultures with senescent, but not non-senescent macrophages. Fibronectin expression was decreased in both co-culture conditions. While significant results were not observed, concentrations of other fibrogenic molecules were consistent with the collagen results. These data demonstrate that senescence in macrophages alters the production of fibrotic molecules from fibroblasts in a SARS-CoV-2 infection model. As collagen and fibronectin expression are generally directly correlated, this suggests that senescence dysregulates fibrogenesis in response to infection with SARS-CoV-2. There is a need to further investigate the mechanisms for these changes.

## Introduction

The Severe Acute Respiratory Syndrome Coronavirus-2 (SARS-CoV-2), since its emergence in 2019, has become a major global pandemic, causing in excess of 900,000 deaths in the USA [1]. The virus can cause respiratory disease with pneumonia [2]. Other commons symptoms include fever, cough, fatigue, aching, loss of smell, sore throat, and nasal congestion [3]. Disease outcomes are worsened in older patients, particularly unvaccinated older patients [4, 5]. Notably, in the early stages of the pandemic the mortality rate for older individuals was reported to be 8 times higher in patients between the ages of 55–64 as compared to younger patients, and 62 times higher in patients over the age of 65 [4]. As individuals age, the amount of senescent cells in the body increase [6]. Cellular senescence is the accumulation of factors resulting in cell growth arrest and resistance to apoptosis [7]. In innate immune cells, aging associated changes in inflammatory response can be associated with impaired immune responses and alterations in fibrogenesis [8–11]. This is especially true in fibrotic lung diseases, including idiopathic pulmonary fibrosis, where the development of pulmonary senescence can result in dysregulated fibrogenesis in fibroblasts [9–11]. The causes for worsened outcomes in older patients with COVID-19 are complex and multifactorial, and we hypothesized that increased amounts of senescent cells represents one cause for these worsened outcomes. Active infection with SARS-CoV-2 has also been associated with increased senescence in cell culture models with lung epithelial cells [12], and the combination of aging- and SARS-CoV-2- dependent senescence, especially among immune cells, has the strong potential to represent a cause for worsened SARS-CoV-2 outcomes [12, 13].

Innate immune cells, including macrophages, are among the first cells to respond to infection with SARS-CoV-2 [14–18]. A strong inflammatory response from macrophages may be the initiating factor for the hypercytokinemia that occurs in some patients [16, 19], [20], [21]. Macrophages also play an important role in orchestrating the influx of other immune cells in COVID-19, including T cells and neutrophils [22, 23]. Senescent macrophages may therefore alter host responses to SARS-CoV-2 [13]. Further, macrophages produce cytokines and chemokines involved in fibrogenesis, fibroblast proliferation, and signaling, and these factors may also be altered due to cellular senescence [24–26]. Alterations in fibrotic molecules, including collagen and fibronectin, may play a role in long-term SARS-CoV-2 damage, especially lung scarring.

We induced senescence in monocyte derived macrophages via exposure to X-ray radiation, and then co-cultured these macrophages in a direct co-culture system consisting of MDM and IMR90 fibroblasts.

## Materials and methods

### Collection of and differentiation of monocyte derived macrophages

Primary human monocytes were collected from healthy volunteers between the ages of 18–35, under University of Memphis IRB protocol 4316. Subjects provided informed consent prior to enrollment. Ten to twelve mL of blood was collected into EDTA-treated vacutainer tubes by venipuncture. Classical Monocytes were enriched using immunomagnetic negative sorting (EasySep Direct Human Monocyte Isolation Kit, StemCell Technologies, Cambridge, MA). This procedure results in a highly pure (>90%) population of classical monocytes [27]. Approximately 2–5 × 10^5^ monocytes were isolated per ml of blood. Monocytes were differentiated to MDM in RPMI + 10% FBS with 10 ng/mL macrophage colony stimulating factor (M-CSF) for 7 days. Senescence was then induced in MDM via 10 Gy irradiation over 10 min using a benchtop irradiator (Cellrad, Precision X-ray, North Branford, CT), and cells were then cultured for 10 days. Non-senescent macrophages were mock-irradiated and treated similarly. Cell culture media with M-CSF was replated every 2–3 days.

### SARS-CoV-2 culture

The SARS-CoV-2 isolate USA-WA1/2020 was used for all experiments. Virus was obtained from BEI Resources (National Institute of Allergy and Infectious Diseases, National Institutes of Health: SARS-Related Coronavirus 2 Isolate USA-WA1/2020, catalog #NR52281). Virus was propagated in Vero-E6 cells (American Type Culture Collection, catalog #VERO C1008) in minimal essential medium + 5% FBS + 5 mM penicillin-streptomycin, and infected at an MOI of 0.1) [28]. Viral titers were measured by plaque assay [29]. All experiments with live SARS-CoV-2 were conducted in a biosafety level 3 laboratory [28]. Virus was used at passage 3.

### Cell culture

5 × 10^4^ non-senescent IMR-90 fibroblasts (ATCC, Manassas, VA) were plated in 24 well plates for 24 h in RPMI + 10% FBS, and incubated at 3% O_2_ and 10% CO_2_. After initial incubation, 5 × 10^4^ senescent or non-senescent macrophages were scraped, centrifuged, resuspended, and added to the culture system, and cells were incubated for an additional 24 h. RPMI + 10% FBS was replaced, and cells were infected with SARS-CoV-2 isolate USA-WA1/2020 at an MOI of 0.5. Cells were incubated with virus for 48 h. Supernatants were collected and frozen at −80 °C for later analysis. 6 replicates were utilized for all experiments.

### Soluble collagen assay

Soluble collagen was assessed in supernatants using the Sircol soluble collagen assay (Biocolor, Carrickfergus, UK), according to manufacturer protocols. In brief, supernatants were treated with isolation and concentration reagent overnight at 4 °C. After initial concentration, supernatants were centrifuged at 12,000 RPM for 10 min, and the supernatant was removed. Sircol dye was added to all tubes and incubated for 30 min. Tubes were again centrifuged at 12,000 RPM for 10 min, and supernatants carefully removed from the tubes. The dye pellet was washed with cold acid salt wash reagent, and centrifuged for 10 min at 12,000 RPM, and supernatants were again removed. Alkali reagent was added to the tubes, and tubes were vortexed to release the dye solution. Supernatants were transferred to 96 well microplates, and absorbance read at 560 nm.

### ELISA assays

Total transforming growth factor beta (TGF-β), fibronectin, fibroblast growth factor (FGF), and interleukin 10 (IL-10) concentrations were assessed via ELISA. Concentrations were assessed using Quantikine (TGF-β, fibronectin) or duoset (FGF, and IL-10) ELISA systems (R&D systems, Minneapolis, MN). ELISAs were run according to manufacturer’s protocols and read at 450 nm. For the TGF-β ELISA, inactive TGF-β was activated via treatment with 1 N HCl for 10 min. The acid was then neutralized via the addition of 1.2 N NaOH. Samples were then processed using standard ELISA techniques. Assay lower limits of detections were TGF-β=31.3 pg/mL, fibronectin=3.13 ng/mL, FGF=15.6 pg/mL, and IL-10=31.3 pg/mL.

### Statistical analysis

Statistical comparisons between samples were made using Graphpad Prism 9 (Graphpad, San Diego, CA). Comparisons were made between uninfected and infected co-cultures using paired T tests, with significance defined as a p value <0.05.

## Results

### Fibrotic protein expression

**Figure 1:**
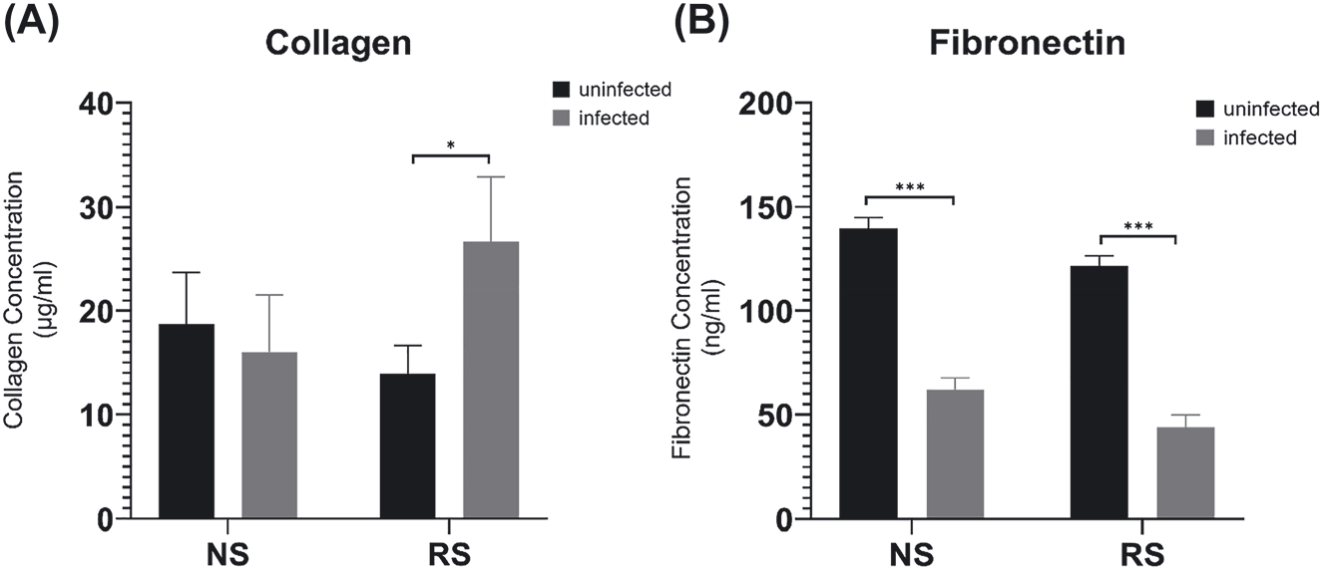
Fibrotic protein expression in co-cultures of senescent/non-senescent macrophages and fibroblasts infected with SARS-CoV-2 USA-WA1/2020. (A) soluble collagen concentration as determined by the Sircol soluble collagen assay. (B) Supernatant fibronectin concentrations as determined by ELISA assay. ^*^p<0.05, ^***^p<0.001 by paired T tests. NS, non-senescent macrophages; RS, radiation-induced senescent macrophages N=6 for all experiments.

We assessed the production of soluble collagen in the macrophage/fibroblast co-culture system in response to SARS-CoV-2 infection. In cultures with non-senescent macrophages, we did not observe any differences in collagen production due to infection with SARS-CoV-2. Contrariwise, infection with SARS-CoV-2 significantly increased collagen production in the co-cultures with senescent macrophages, an approximately 2-fold increase (Figure 1A). We also examined supernatant concentrations of fibronectin, another component of the extracellular matrix (Figure 1B) [30, 31]. We observed that infection with SARS-CoV-2 significantly decreased fibronectin expression in co-cultures with both non-senescent and senescent macrophages.

### Pro-fibrotic cytokine expression

**Figure 2:**
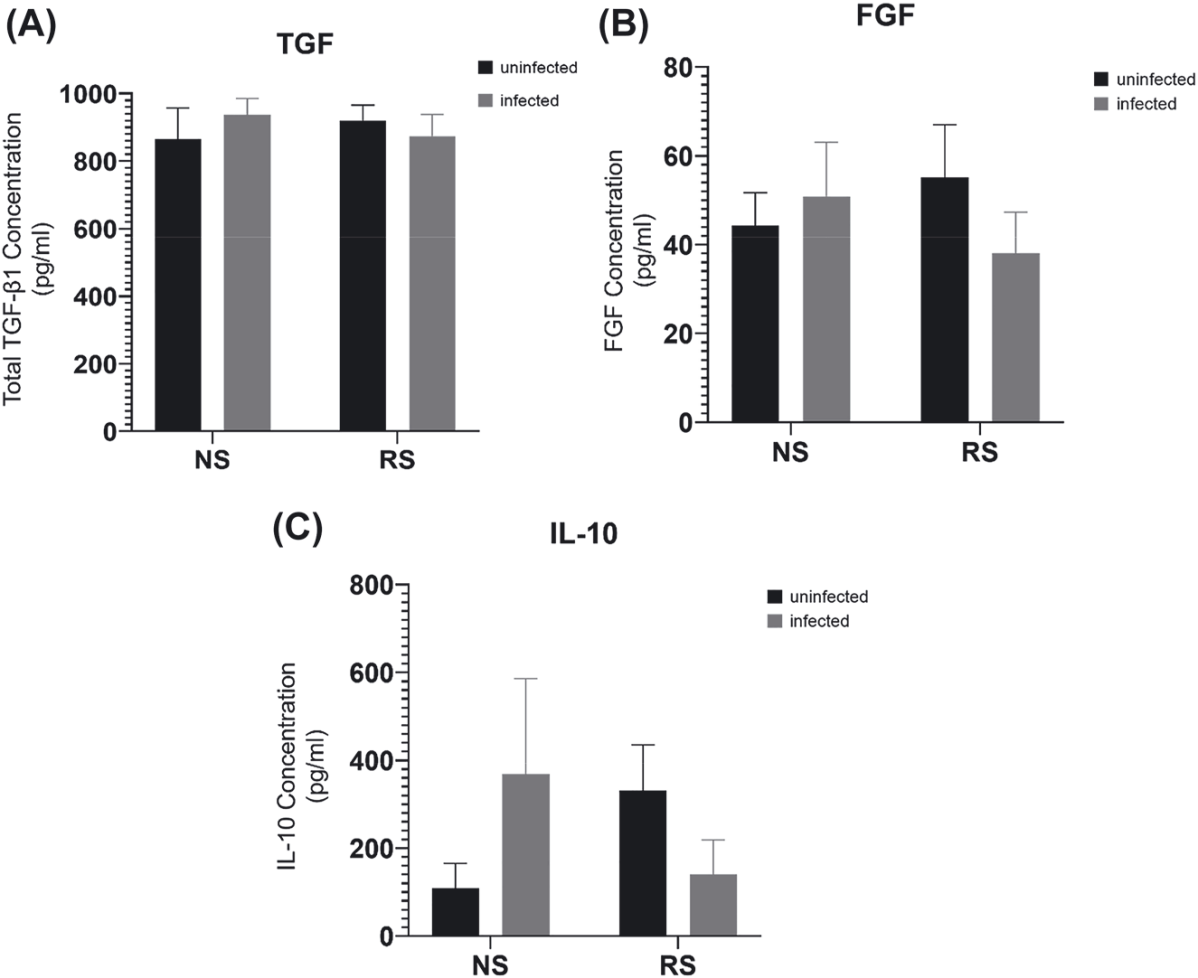
Fibrogenic protein expression in co-cultures of senescent/non-senescent macrophages and fibroblasts infected with SARS-CoV-2 USA-WA1/2020. ELISA assays for (A) total TGF-β1, (B) FGF, and (C) IL-10. NS, non-senescent macrophages; RS, radiation induced senescent macrophages N=6 for all experiments.

We finally assessed the expression of TGF-β, FGF, and IL-10, cytokines which are associated with fibrogenesis, and which can be produced by macrophages (Figure 2) [32–35]. Surprisingly, we did not observe any differences in total TGF-β in cell culture supernatants, importantly the ELISA that we utilized was unable to differentiate between active and total TGF-β (Figure 2A). FGF expression was non-significantly downregulated in senescent, but not non-senescent co-cultures (Figure 2B). Finally, when we examined IL-10, while no significant differences were observed, we observed a non-significant trend that infection increased IL-10 production in non-senescent macrophage co-cultures, while it was decreased in senescent macrophage co-cultures (Figure 2C).

## Discussion

Cellular senescence increases as individuals age, and results in increased inflammation and cellular damage [13, 36], [37], [38]. We induced senescence in macrophages using X-ray irradiation and assessed how senescent macrophages and non-senescent fibroblasts interact to produce fibrotic molecules including collagen and fibronectin. Our data shows that senescent macrophages altered the production of an important fibrotic molecule from fibroblasts in our co-culture system when infected with SARS-CoV-2, supporting that increased immune cell senescence may be one of the causes for the worsened outcomes that occur in older patients infected with SARS-CoV-2.

We observed no alterations in soluble collagen concentrations due to infection in the non-senescent macrophage co-cultures, while collagen production was significantly increased in response to infection (approximately 2-fold) in the co-culture with senescent macrophages and non-senescent fibroblasts (Figure 1A). While we assessed soluble and not matrix collagen, over time soluble collagen is deposited in the extracellular matrix. *In vivo*, extracellular matrix collagen is produced as part of the tissue repair and remodeling that occurs after an acute infection [39, 40], and the significant increase that we observed in the senescent macrophage co-culture supports the hypothesis that senescent macrophages alter fibrogenesis from fibroblasts. This may represent a cause of worsened outcomes in older patients infected with SARS-CoV-2. We also observed that active infection with SARS-CoV-2 significantly decreased soluble fibronectin in both non-senescent and senescent macrophage co-cultures (Figure 1B). In the extracellular matrix, collagen and fibronectin crosslink to form the main body of the extracellular matrix [41]. As such, collagen and fibronectin expression are generally directly correlated [42]. We instead observed that in the senescent macrophage co-cultures, infection with SARS-CoV-2 increased collagen and decreased fibronectin expression. This dysregulation suggests a potential cause of worsened outcomes in older patients infected with SARS-CoV-2, as well as suggests a potential target for improving outcomes in this patient population.

Surprisingly, we did not observe any significant differences in total TGF-β or FGF due to infection with SARS-CoV-2 in either the non-senescent or senescent co-culture systems (Figure 2A and B). Importantly, the ELISA that we utilized was unable to distinguish between inactive and active TGF-β, instead only assessing total TGF-β. As TGF-β is secreted in its inactive state, and is cleaved to become active, we are unable to definitively state if senescent macrophages influence TGF-β activation, only its total expression in our co-culture system [43]. While the results were non-significant, the decrease in soluble FGF concentrations in the infected co-cultures with senescent macrophages is consistent with findings from others showing that FGF downregulates collagen production and upregulates collagenase production, although larger conclusions are difficult to make based on the lack of significant findings in our study [44–46].

We also did not observe significant differences in the expression of IL-10, although we did observe non-significant trends. Low IL-10 expression has been associated with increased fibrogenesis in some parasite infections [47]. In our study, we observed non-significant increases in IL-10 due to infection in non-senescent macrophage co-cultures, and a non-significant decrease in senescent macrophage co-cultures, which is again consistent with the collagen data that we observed. These findings differ from observations from others showing that senescent macrophages produce elevated IL-10 [48, 49].

Additional studies are necessary to further elucidate the mechanisms by which infection with SARS-CoV-2 dysregulates fibrogenesis, both *in vitro* and *in vivo*. This may include the utilization of transwell inserts to quantify both paracrine and juxtacrine interactions between senescent macrophages and fibroblasts. It also will be necessary to assess these interactions *in vivo*, by adoptively transferring senescent or non-senescent macrophages into mice prior to infection with SARS-CoV-2. Finally, SARS-CoV-2 has continued to evolve over the course of the pandemic. Mutations in the virus may influence the host immune response, and assessing how viral variants influence senescence-dependent fibrogenesis is a high priority.

Our data shows that in a co-culture system with an immortalized non-senescent fibroblast cell line, and either senescent or non-senescent macrophages, co-cultures with senescent macrophages saw a significant increase in soluble collagen concentrations in response to infection with SARS-CoV-2. Increased collagen concentrations suggest that senescent macrophages produce paracrine or juxtacrine factors that differentially increase fibrogenesis as compared to non-senescent macrophages. As cellular senescence increases in older individuals, this may represent one of the causes of worsened outcomes in older patients infected with SARS-CoV-2, as well as suggests that these individuals may suffer long term outcomes related to increased scarring and fibrosis.
